# Long-term trends and survival analysis of esophageal and gastric cancer in Yangzhong, 1991-2013

**DOI:** 10.1371/journal.pone.0173896

**Published:** 2017-03-13

**Authors:** Zhaolai Hua, Xianzhi Zheng, Hengchuan Xue, Jianming Wang, Jun Yao

**Affiliations:** 1 Department of Epidemiology, Yangzhong Cancer Research Institute, Yangzhong, China; 2 Department of Epidemiology, School of Public Health, Nanjing Medical University, Nanjing, China; 3 Department of Thoracic Surgery, People’s Hospital of Yangzhong, Yangzhong, China; 4 The Innovation Center for Social Risk Governance in Health, School of Public Health, Nanjing Medical University, Nanjing, China; 5 Department of Gastroenterology, Zhenjiang First People’s Hospital, Zhenjiang, China; Peter MacCallum Cancer Centre, AUSTRALIA

## Abstract

**Objective:**

To describe the long-term trends of the incidence, mortality and survival of upper digestive tract cancers in a high-risk area of China.

**Methods:**

We extracted esophageal and gastric cancer cases diagnosed from 1991 to 2013 through the Yangzhong Cancer Registry and calculated the crude and age-standardized incidence and mortality rates. Cancer trends were calculated using the Joinpoint Regression Program and were reported using the annual percentage change (APC). The cancer-specific survival rates were evaluated and compared between groups using the Kaplan-Meier method and log-rank test.

**Results:**

The age-standardized incidence rate of esophageal cancer declined from 107.06 per 100,000 person-years (male: 118.05 per 100,000 person-years; female: 97.42 per 100,000 person-years) in 1991 to 37.04 per 100,000 person-years (male: 46.43 per 100,000 person-years; female: 27.26 per 100,000 person-years) in 2013, with an APC of -2.5% (95% confidence interval (CI): -3.4%, -1.5%) for males and -4.9% (95% CI:-5.8%, -3.9%) for females. The age-standardized incidence rate of gastric cancer was 165.11 per 100,000 person-years (male: 225.39 per 100,000 person-years; female: 113.34 per 100,000 person-years) in 1991 and 53.46 per 100,000 person-years (male: 76.51 per 100,000 person-years; female: 32.43 per 100,000 person-years) in 2013, with the APC of -3.6% (95% CI: -4.5%, -2.7%) for males and -4.8% (95% CI: -5.7%, -3.9%) for females. The median survival time was 3.0 years for patients with esophageal or gastric cancer. Cancer cases detected after 2004 had a better prognosis.

**Conclusions:**

The age-standardized incidence rates of both esophageal and gastric cancer continuously decreased since 1991 through 2013, whereas the mortality rate remained stable before 2004 and significantly declined following the massive endoscopic screening program initiated in 2004. The survival probability of patients with esophageal and gastric cancer has improved obviously in recent decades.

## Introduction

The two most common upper digestive tract cancers worldwide, esophageal cancer and gastric cancer, are the sixth and third leading cause of cancer-related death, respectively [1]. Since the middle of the 20^th^ century, a steady decline in upper digestive tract cancer incidence has been observed in the majority of more developed countries in Northern America and Europe [2–4]. Similar decreasing trends have also been noted in areas with historically high rates, including Ukraine, Colombia, Ecuador, Japan, China and Korea, but the disease burden remains heavy in some developing countries [5, 6].

A long-term trend analysis from in China revealed that the all-cancer incidence rates were stable during 2000 through 2011 for males, whereas they increased significantly among females. In contrast, the mortality rates since 2006 have declined significantly for both males and females [5]. The four most common cancers diagnosed in China are lung, gastric, liver, and esophageal, accounting for 57% of all cancers diagnosed in China, compared with 18% in the United States [5, 7]. Gastric, esophageal, and liver cancers were identified as leading causes of cancer-related death [5]. Malignancies of both esophageal and gastric cancers have a particularly poor prognosis and lower survival rate as they typically cause no symptoms and thus are diagnosed with distant metastasis [8, 9].

Yangzhong is an island located in the middle of the Yangtze River, China. It consists of a group of small islands with a special geographical environment and life style [10]. Yangzhong has been reported as a high-risk area for both esophageal cancer and gastric cancer since the 1970s. In 1985, the Yangzhong Cancer Institute was established. In 1991, the Yangzhong Cancer Registry was developed [10]. Since 2004, massive screening and intervention programs for digestive tract caners have been implemented [11], but their effects on the morbidity and mortality of esophageal and gastric cancers have not been thoroughly investigated. Like other health indices, information on survival statistics is an important component in monitoring cancer control activities, which may suggest possible reasons for changes and provide targets for the improvement. Based on the local cancer registry database, we performed the current study to describe the long-term trends of the incidence, mortality and survival of upper digestive tract cancers in Yangzhong.

## Methods

### Ethics statement

This project has been approved by the Institutional Review Board of Nanjing Medical University. Written informed consent was obtained from all participants.

### Data source

Yangzhong is a county-level city in eastern China, with a population of 280,000 and an area of 327 km^2^ at the end of 2015. The Yangzhong Cancer Mortality Registry was established in 1985 and is organized and maintained by the Yangzhong Cancer Research Institute (affiliated with Yangzhong People’s Hospital). In 1990, case reports of both cancer occurrence and death to the Yangzhong Cancer Registry became compulsory, and the registry has functioned well since 1991. The cancer registry covers the entire local population. Cancer cases are reported to the registry from multiple sources, including local hospitals, community health centers and village clinics. A standard notification card is used to report the information of cancer cases, including patient’s demographic characteristics (name, date of birth, sex, address and occupation) and disease characteristics (type of cancer, cancer site, date of diagnosis, pathology, and treatment). Patients reported to Yangzhong Cancer Registry were followed to confirm their diagnosis and survival status. Both active case searching and passive case reporting methods were adopted. For passive follow-up, death information about patients with esophageal or gastric cancer was extracted from death certificates in the vital statistical section of Yangzhong Center for Disease Control and Prevention. Patients without death information were considered to be “alive” until the end time of our research. In addition, mortality data were matched with the cancer incidence database. Active follow-up was necessary in the absence of reliable health information. Home visits or postal/telephone enquiries were performed to confirm the essential information. We extracted data from 1991 to 2013 based on the registry database. All cancer cases identified as codes C15 (esophageal cancer) or C16 (gastric cancer) from the 10^th^ revision of the International Classification of Disease (ICD-10) were included in the analysis [12]. The information of death, living status or loss to follow-up were updated through December 31, 2015. Patients that were alive at the closing date, lost to follow-up or died by other causes were considered censored [13, 14].

### Quality control

Data reported to the Cancer Registry were checked for eligibility and validity before being entered into the database. Quality was assessed based on the criteria of the International Agency for Research on Cancer/International Association of Cancer Registries (IARC/IACR). Additionally, data sorting, checking and evaluation were performed using relevant software, including Excel and IARC-crgTools [15, 16]. The proportion of morphological verification (MV %), percentage of cancer cases identified with death certification only (DCO %), mortality to incidence ratio (MI), percentage of uncertified cancer (UB %), and percentage of cancer with undefined or unknown primary site (secondary) (CPU %) were used to evaluate the completeness, validity and reliability of the data [17]. In the current study, the overall indicators of MV %, DCO %, and MI ratio were 71.23%, 2.98% and 0.65, respectively. The UB % and CPU % were less than 5% for both esophageal and gastric cancers.

### Statistical analyses

The annual age-standardized rate and age-specific rate were calculated from 1991 through 2013. The age-adjusted rates represent a weighted average of the age-specific rates in which the weights are the proportions of persons in the corresponding age group of the standard population. This method reduced the potential confounding effect caused by age. We calculated the world age-standardized rate according to Segi’s world standard population [18]. Cancer trends were calculated using the Joinpoint Regression Program and were reported using the annual percentage change (APC). Joinpoint Regression Program 4.3.1.0 was downloaded from the website of the National Cancer Institute (NCI, MD, USA). The Kaplan-Meier estimator was used to plot the survival curves. The 3-year cancer-specific survival rates (proportion of patients alive at the specified time) were calculated. Survival analysis between groups was performed using the log-rank test [19]. All statistical analyses were performed using SPSS 18.0 or STATA 10.0 software.

## Results

### Esophageal cancer

**Fig 1:**
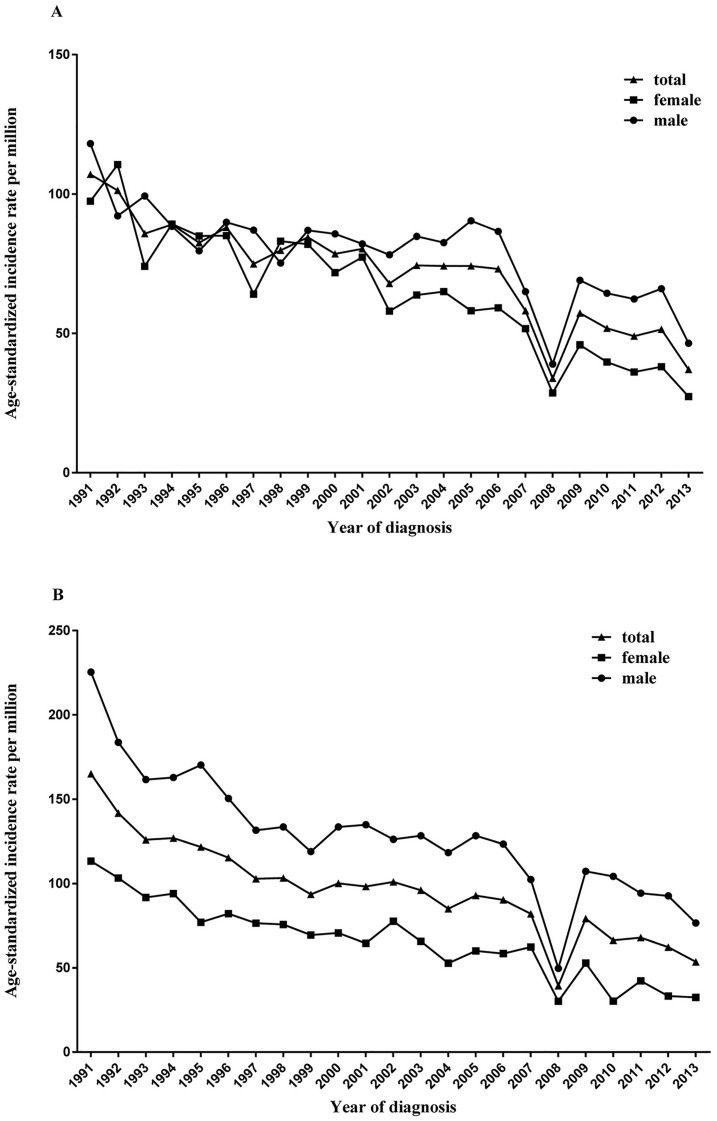
Age-adjusted incidence rates of esophageal and gastric cancers in Yangzhong. A: esophageal cancer; B: gastric cancer.

**Fig 2:**
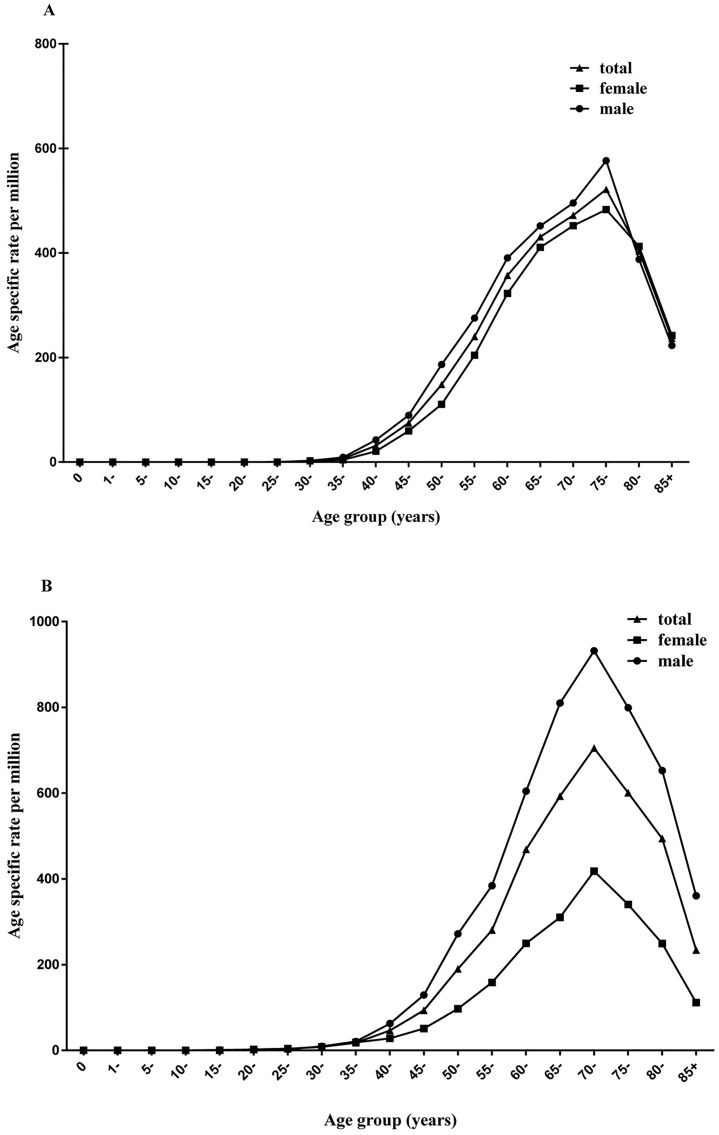
Age-specific incidence rates of esophageal and gastric cancer in Yangzhong. A: esophageal cancer; B: gastric cancer.

From 1991 to 2013, 6493 esophageal cancer cases were identified in Yangzhong County. Among them, 3446 (53.1%) were men and 3047 (46.9%) were women. The crude incidence rate of esophageal cancer was relatively stable during the study period, but the age-standardized incidence significantly decreased from 107.06 per 100,000 person-years (male: 118.05 per 100,000 person-years; female: 97.42 per 100,000 person-years) in 1991 to 37.04 per 100,000 person-years (male: 46.43 per 100,000 person-years; female: 27.26 per 100,000 person-years) in 2013, with an APC of -2.5% (95% CI: -3.4%, -1.5%) for males (P <0.01) and -4.9% (95% CI: -5.8%, -3.9%) for females (P <0.01) (Tables 1 and 2; Fig 1A). The age-specific rates were relatively low in populations younger than 40 years and increased with age, reaching the peak at 75 years of age (Fig 2A). The age-specific rate was higher in males than in females for each age group.

**Table 1:** Incidence rate of esophageal and gastric cancer in Yangzhong, 1991–2013.

Cancers	Year	Male			Female			Total			
		N	CR	ASR	N	CR	ASR	N	CR	ASR
Esophageal cancer	1991	159	117.60	118.05	150	108.15	97.42	309	112.82	107.06
	1992	131	96.96	92.13	173	124.91	110.54	304	111.11	101.28
	1993	145	107.40	99.28	122	88.21	74.06	267	97.69	85.75
	1994	137	101.55	88.36	152	110.06	89.14	289	105.86	89.03
	1995	125	92.72	79.61	151	109.49	84.92	276	101.20	82.51
	1996	147	109.12	89.86	158	114.73	85.04	305	111.96	87.97
	1997	149	110.68	87.04	117	85.08	64.09	266	97.75	74.91
	1998	132	98.12	75.22	157	114.33	83.09	289	106.31	79.81
	1999	157	116.79	86.92	162	118.14	81.97	319	117.48	84.46
	2000	159	118.35	85.66	142	103.66	71.80	301	110.94	78.54
	2001	155	115.43	82.08	162	118.39	77.34	317	116.93	80.39
	2002	154	114.81	78.20	129	94.48	57.98	283	104.56	67.88
	2003	163	121.52	84.74	138	101.07	63.74	301	111.21	74.37
	2004	164	122.10	82.55	140	102.48	64.99	304	112.21	74.19
	2005	182	135.31	90.37	132	96.56	58.07	314	115.78	74.17
	2006	184	136.28	86.55	138	100.08	59.13	322	117.99	73.13
	2007	146	107.85	64.99	120	86.64	51.69	266	97.13	58.07
	2008	86	63.35	38.96	76	54.69	28.59	162	58.97	33.86
	2009	156	113.31	69.03	111	78.42	45.84	267	95.63	57.22
	2010	160	115.32	64.40	106	74.21	39.7	266	94.47	51.79
	2011	154	111.50	62.30	104	73.08	36.14	258	92.01	49.00
	2012	174	126.17	66.02	118	82.55	38.03	292	103.97	51.44
	2013	127	91.92	46.43	89	62.05	27.26	216	76.71	37.04
Gastric cancer	1991	300	221.90	225.39	171	123.29	113.34	471	171.97	165.11
	1992	258	190.97	183.73	165	119.13	103.24	423	154.61	141.72
	1993	235	174.07	161.61	157	113.52	91.72	392	143.43	125.9
	1994	247	183.09	162.87	163	118.03	93.97	410	150.18	126.86
	1995	264	195.83	170.26	140	101.52	76.94	404	148.14	121.69
	1996	242	179.64	150.44	150	108.92	82.11	392	143.89	115.37
	1997	222	164.92	131.57	138	100.35	76.47	360	132.29	102.85
	1998	225	167.27	133.51	143	104.14	75.62	368	135.38	103.29
	1999	209	155.48	118.93	140	102.10	69.36	349	128.53	93.54
	2000	242	180.13	133.52	143	104.40	70.6	385	141.90	100.12
	2001	251	186.93	134.88	136	99.40	64.57	387	142.75	98.19
	2002	248	184.90	126.2	170	124.51	77.59	418	154.44	101
	2003	252	187.88	128.35	136	99.61	65.71	388	143.35	96.05
	2004	228	169.76	118.35	112	81.99	52.75	340	125.50	84.99
	2005	260	193.31	128.28	129	94.37	60.01	389	143.44	92.87
	2006	265	196.27	123.33	133	96.46	58.45	398	145.84	90.27
	2007	228	168.43	102.39	153	110.48	62.31	381	139.12	81.98
	2008	114	83.99	49.59	71	51.09	30.12	185	67.35	39.26
	2009	237	172.15	107.25	137	96.79	52.78	374	133.95	79.16
	2010	250	180.19	104.21	84	58.81	30.22	334	118.62	66.32
	2011	236	170.88	94.3	120	84.33	42.22	356	126.96	67.86
	2012	240	174.04	92.64	93	65.06	33.24	333	118.57	62.26
	2013	206	149.10	76.51	94	65.54	32.43	300	106.54	53.46

Abbreviations: N, number of cases; CR, crude rate; ASR, age-standardized rate.

**Table 2:** Joinpoint analysis of esophageal and gastric cancers in Yangzhong, 1991–2013.

Cancers	Gender	Incidence			Mortality		
		Period	APC(95% CI)	Tendency P-value	Period	APC(95% CI)	Tendency P-value
Esophageal cancer	Total	1991–2013	-3.5(-4.3, -2.7)	<0.01	1991–1993	47.5(-14.4, 154.1)	0.1
					1993–2004	-2.9(-5.8, 0.5)	0.1
					2004–2010	-17.7(-26.2, -8.3)	<0.01
					2010–2013	16.8(-11.4, 53.9)	0.2
	Male	1991–2013	-2.5(-3.4, -1.5)	<0.01	1991–1993	41.9(-23, 161.5)	0.2
					1993–2004	-2.3(-5.6, 1.1)	0.2
					2004–2010	-15(-24.2, -4.7)	<0.01
					2010–2013	15.3(-13.6, 53.8)	0.3
	Female	1991–2013	-4.9(-5.8, -3.9)	<0.01	1991–2000	1.5(-4.8, 8.1)	0.6
					2000–2013	-11.9(-15.5, -8.1)	<0.01
Gastric cancer	Total	1991–2013	-4.0(-4.8, -3.2)	<0.01	1991–2001	1.1(-2.8, 5.2)	0.6
					2001–2011	-13.8(-18.3, -8.9)	<0.01
					2011–2013	34.4(-26.6, 146.1)	0.3
	Male	1991–2013	-3.6(-4.5, -2.7)	<0.01	1991–1993	38.5(-7.9, 108.3)	0.1
					1993–2002	-1.9(-4.9, 1.2)	0.2
					2002–2011	-13.5(-16.9, -10)	<0.01
					2011–2013	40(-1.1, 98.2)	0.1
	Female	1991–2013	-4.8(-5.7, -3.9)	<0.01	1991–1999	2.4(-4.9, 10.2)	0.5
					1999–2013	-11(-14.3, -7.6)	<0.01

Abbreviations: APC, annual percentage change; CI, confidence interval.

**Fig 3:**
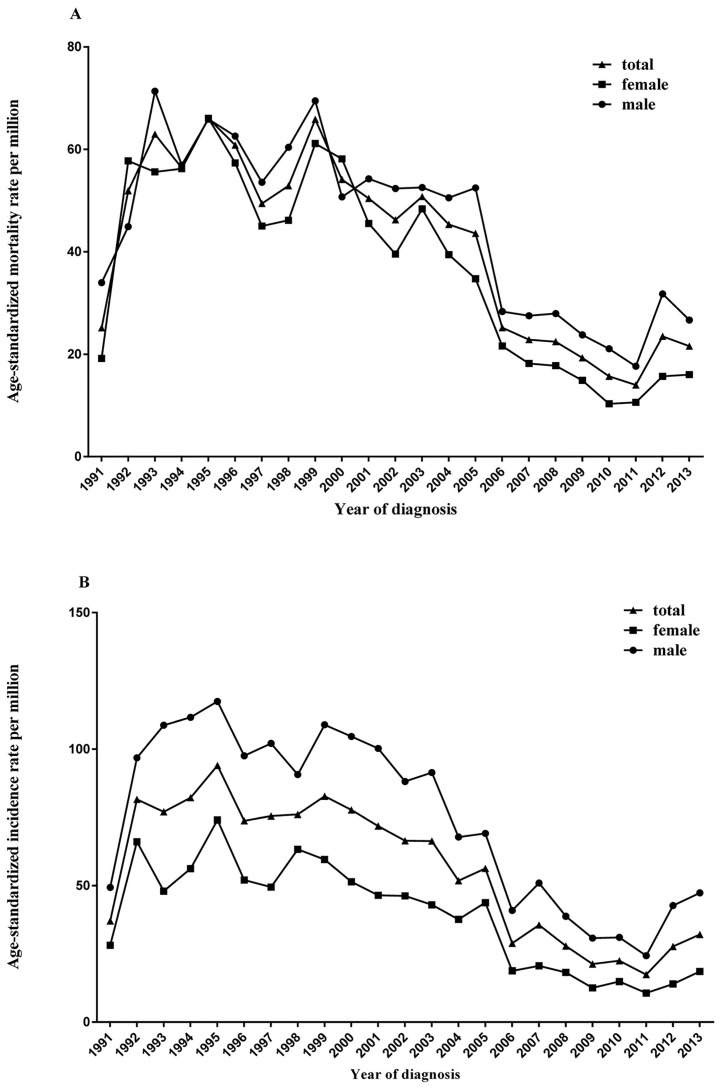
Age-adjusted mortality rates of esophageal and gastric cancers in Yangzhong. A: esophageal cancer; B: gastric cancer.

The age-standardized mortality rate for esophageal cancer increased from 1991 (25.15 per 100,000 person-years) to 1993 (62.94 per 100,000 person-years), remained stable from 1994 (56.38 per 100,000 person-years) to 2003 (50.78 per 100,000 person-years) and then decreased from 2004 (45.32 per 100,000 person-years) to 2011 (13.98 per 100,000 person-years), with a significant APC of -17.7% (95% CI: -26.2%, -8.3%; P <0.01) (Tables 2 & 3; Fig 3A).

**Table 3:** Mortality rate of esophageal and gastric cancers in Yangzhong, 1991–2013.

Cancers	Year	Male			Female			Total		
		N	CR	ASR	N	CR	ASR	N	CR	ASR
Esophageal cancer	1991	43	31.81	33.96	30	21.63	19.17	73	26.65	25.15
	1992	63	46.63	44.91	92	66.43	57.73	155	56.65	51.9
	1993	104	77.04	71.36	93	67.24	55.6	197	72.08	62.94
	1994	87	64.49	56.91	96	69.51	56.22	183	67.03	56.38
	1995	103	76.41	65.87	118	85.56	66.03	221	81.04	66.05
	1996	103	76.46	62.57	106	76.97	57.35	209	76.72	60.82
	1997	91	67.60	53.56	90	65.45	45.02	181	66.51	49.41
	1998	104	77.31	60.39	87	63.36	46.14	191	70.26	52.89
	1999	125	92.99	69.45	123	89.70	61.13	248	91.33	65.85
	2000	92	68.48	50.72	120	87.61	58.14	212	78.14	54.11
	2001	102	75.96	54.24	96	70.16	45.53	198	73.04	50.39
	2002	103	76.79	52.36	91	66.65	39.56	194	71.68	46.23
	2003	101	75.30	52.56	108	79.10	48.37	209	77.22	50.78
	2004	100	74.45	50.53	88	64.42	39.46	188	69.39	45.32
	2005	107	79.55	52.45	76	55.60	34.73	183	67.48	43.58
	2006	62	45.92	28.32	53	38.44	21.61	115	42.14	25.17
	2007	62	45.80	27.52	48	34.66	18.18	110	40.17	22.86
	2008	61	44.94	27.93	47	33.82	17.77	108	39.32	22.46
	2009	55	39.95	23.77	40	28.26	14.88	95	34.02	19.3
	2010	52	37.48	21.07	30	21.00	10.33	82	29.12	15.68
	2011	47	34.03	17.67	32	22.49	10.6	79	28.17	13.98
	2012	82	59.46	31.76	49	34.28	15.69	131	46.65	23.5
	2013	73	52.84	26.65	55	38.35	16.02	128	45.46	21.57
Gastric cancer	1991	63	46.60	49.33	43	31.00	28.11	106	171.97	37.09
	1992	134	99.19	96.83	109	78.70	66.07	243	154.61	81.59
	1993	158	117.04	108.79	83	60.01	47.97	241	143.43	77.06
	1994	165	122.31	111.66	101	73.13	56.24	266	150.18	92.25
	1995	183	135.75	117.45	133	96.44	74.05	316	148.14	94.02
	1996	154	114.32	97.58	96	69.71	52.06	250	143.89	73.73
	1997	170	126.29	102.1	94	68.36	49.47	264	132.29	75.50
	1998	157	116.71	90.69	119	86.66	63.35	276	135.38	76.10
	1999	187	139.12	108.96	120	87.51	59.56	307	128.53	82.75
	2000	188	139.94	104.66	108	78.85	51.44	296	141.90	77.75
	2001	188	140.01	100.29	97	70.89	46.42	285	142.75	71.85
	2002	170	126.74	88.17	101	73.98	46.25	271	154.44	66.43
	2003	177	131.96	91.41	88	64.45	42.97	265	143.35	66.36
	2004	131	97.54	67.82	79	57.83	37.68	210	125.50	51.81
	2005	141	104.83	69.13	97	70.96	43.72	238	143.44	56.28
	2006	88	65.18	40.87	42	30.46	18.77	130	145.84	28.87
	2007	111	82.00	50.96	52	37.55	20.64	163	139.12	35.58
	2008	91	67.04	38.79	45	32.38	18.2	136	67.35	27.88
	2009	68	49.39	30.78	36	25.43	12.5	104	133.95	21.24
	2010	74	53.34	31.04	40	28.00	14.84	114	118.62	22.48
	2011	60	43.44	24.34	34	23.89	10.59	94	126.96	17.37
	2012	112	81.22	42.67	39	27.28	13.96	151	118.57	27.70
	2013	127	91.92	47.34	56	39.04	18.56	183	106.54	32.11

Abbreviations: N, number of cases; CR, crude rate; ASR, age-standardized rate.

### Gastric cancer

From 1991 through 2013, 8537 patients were diagnosed with gastric cancer, of which 5459 (63.9%) were men and 3078 (36.1%) were women. The crude incidence rate of gastric cancer declined throughout the study period, and the age-standardized incidence rate declined significantly from 165.11 per 100,000 person-years (male: 225.39 per 100,000 person-years; female: 113.34 per 100,000 person-years) in 1991 to 53.46 per 100,000 person-years (male: 76.51 per 100,000 person-years; female: 32.43 per 100,000 person-years) in 2013. The APC was -3.6% (95% CI: -4.5%, -2.7%) for males (P <0.01) and -4.8% (95% CI: -5.7%, -3.9%) for females (P <0.01), according to the age-standardized incidence rate (Tables 1 and 2; Fig 1B). The age-specific rates were relatively low in populations younger than 45 years and then increased significantly with age, reaching the peak at 70 years (Fig 2B). Similar to esophageal cancer, the age-specific rate was higher in males than in females in each age group.

The age-standardized mortality rate for gastric cancer fluctuated during 1992 (81.59 per 100,000 person-years) and 2001 (71.85 per 100,000 person-years), and then decreased significantly from 2002 (66.43 per 100,000 person-years) to 2011 (17.37 per 100,000 person-years), with an APC of -13.5% (95% CI: -16.9%, -10.0%; P <0.01) (Tables 2 and 3; Fig 3B).

### Survival analysis

**Fig 4:**
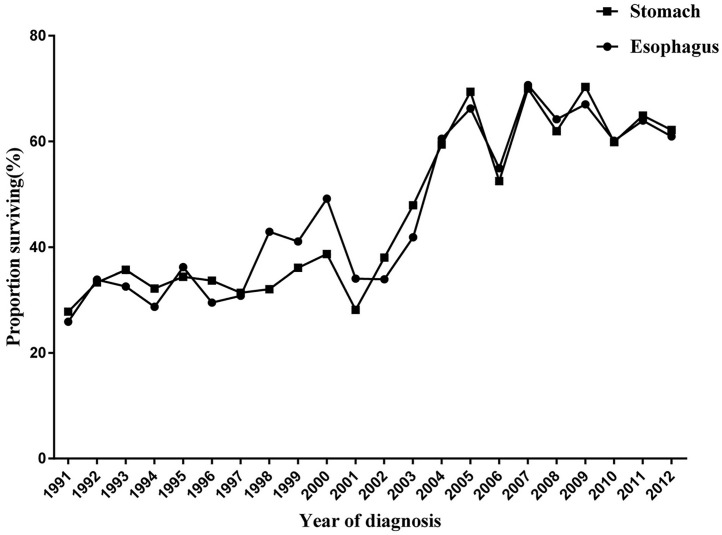
Three-year survival rates of esophageal and gastric cancers in Yangzhong.

All cancer patients were followed for survival status and cause of death. Through December 31, 2015, 3940 (60.7%) of the 6493 esophageal cancer patients and 5228 (61.2%) of 8537 gastric cancer patients died of cancer-related causes. The median survival time was 2.99 years for patients with esophageal cancer and 2.92 years for patients with gastric cancer. The 3-year survival rate for esophageal cancer increased from 25.89% (95% CI: 21.15%, 30.87%) in 1991 to 60.88% (95% CI: 55.02%, 66.21%) in 2012 (P for trend <0.001). The 3-year survival rate for gastric cancer increased from 27.81% (95% CI: 23.84%, 31.91%) in 1991 to 62.13% (95% CI: 56.69%, 67.10%) in 2012 (P for trend <0.001) (Fig 4).

**Fig 5:**
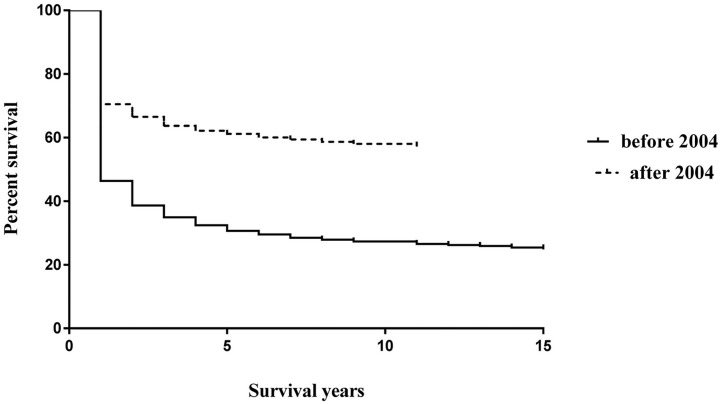
Survival curves of upper digestive tract cancer before and after 2004.

As a massive endoscopic screening program was initiated in 2004 in Yangzhong, we compared the survival rates of two time periods (1991–2003 and 2004–2012). As shown in Fig 5, a significantly positive prognosis was observed among patients diagnosed after 2004 (Log-rank test: P <0.001).

## Discussion

Esophageal cancer and gastric cancer are two common cancers in China. Despite the declining trends of them worldwide [5], the absolute number of cases in China is enormous, partly due to the population size and age [20, 21]. In this longitudinal study, we observed declining trends in the incidence and mortality rates of both esophageal and gastric cancers. Survival analyses showed that the survival probability of patients with esophageal and gastric cancer in Yangzhong has significantly improved during the most recent decades.

The incidence and mortality rates of upper digestive tract cancers were not entirely consistent in different areas. Esophageal cancer is a relatively rare form of cancer but some countries like China, Iran, India, and Japan have a higher incidence than others [22]. There is considerable variation in the incidence of gastric cancer among different geographic regions in the world, with nearly two-thirds occurring in developing countries [22]. In Asia, the highest incidence of gastric cancer has been reported from some eastern countries such as China, Korea and Japan and the low incidence rates are found in south Asia [5, 22]. Even in China, there is a considerable variation in the incidence of both esophageal cancer and gastric cancer. For example, the estimated new cases of esophageal cancer was 364,100 in rural areas which was significantly higher than that in urban areas (113,800) [5]. Possible reasons for the variation in incidence and mortality include racial differences, environment, diet, cooking habits, behaviors, effectiveness of prevention, early detection and management of cancer cases in different areas [1, 21, 23–26]. The incidence rate in the study area increased sharply among people aged more than 40 years and reached to a peak during the age group of 70–75 years. This suggests that we can select residents aged 40–69 years as candidates for cancer screening.

Previous studies in Yangzhong have revealed that hot-temperature food items, pork braised in brown sauce, old stocked rice intake, pickled vegetables, and drinking river water increase the risk of esophageal cancer or gastric cancer [25, 27]. In recent decades, Yangzhong has undergone rapid economic growth. Living conditions and standards have significantly improved. Everyone has access to safe tap water. The dietary pattern has undergone great changes, and the nutritional status of the local population has also improved. These factors may contribute to the decrease in incidence of upper digestive tract cancers. But the rapid growth of the population, aging, increased tobacco smoking, and H. pylori infection may also contribute to the burden of gastrointestinal cancer [21, 28].

The good to excellent prognosis of patients with upper digestive tract cancers in the United States is mainly due to early detection and early treatment [28, 29]. Although prevention efforts are critical to reduce the long-term burden of cancer, the effects of intervention may not be seen immediately [5]. Therefore, facilitating the early diagnosis of cancer and improving access to and availability of optimal treatments may have the greatest potential to have a significant impact on the existing burden of cancer in China [30]. Many studies have shown that endoscopic screening is effective for the early detection of upper digestive tract cancers, thus reducing incidence and fatality [31]. In 2004, an early detection and treatment program was initiated in China [32]. As a pilot site, Yangzhong was selected to implement a population-based endoscopic screening program for early detection and treatment of esophageal and gastric cancer. One of our previous studies revealed a significant positive effect on the survival of patients diagnosed through massive endoscopic screening [11]. In this study, by comparing the age of diagnosis before and after 2004, we found a mean age shift from 65.09 years to 62.11 years (P <0.001), which may be partially attributed to the screening program. However, caution is required in interpreting the long-term survival rate in Fig 5, as the follow-up time in this study was not longer enough to allow a precise estimation of survival beyond five-years.

The present study has several limitations. First, we lack necessary information to analyze the variation in subtypes of esophageal and gastric cancers. Due to the retrospective nature of this study, we failed to obtain all the necessary variables to control the bias in estimating the survival rate. Second, we need to be cautious in interpreting the results regarding trends of cancer occurrence and in linking the longitudinal trends to the implementation of the screening program. Variation in exposure to environmental risk factors or changes in lifestyle across this period may also play an important role. Third, the intention of screening is to diagnose a disease earlier than it would be without screening. However, the leading time bias may contribute to the increase in survival percentage after 2004. This is an important factor when evaluating the effectiveness of the endoscopic screening program. Fourth, The possible "period effects", which would have been associated with the improved completeness of the registries over time or the introduction of new diagnostic tools in recent years, could not be ruled out. However, such period effects might have contributed to artificially increased trends rather than decreasing trends as observed in this study.

## Conclusion

The age-standardized incidence rates of both esophageal and gastric cancer continuously decreased from 1991 through 2013, whereas the mortality rate remained stable before 2004 and significantly declined following the massive endoscopic screening program initiated in 2004. The survival probability of patients with esophageal and gastric cancer has improved in recent decades. Information from this study provides a better understanding of survival differences that are influenced by changing prevention and treatment strategies.
